# Safety evaluation of a buffer used in the lyophilization of canine platelets: type I hypersensitivity reaction in dogs caused by bovine serum albumin

**DOI:** 10.3389/fvets.2024.1344037

**Published:** 2024-02-12

**Authors:** Hee-Jae Choi, Mu-Young Kim, Hyun-Jung Han

**Affiliations:** ^1^Department of Veterinary Emergency and Critical Care, College of Veterinary Medicine, Konkuk University, Seoul, Republic of Korea; ^2^Department of Veterinary Surgery, College of Veterinary Medicine, Konkuk University, Seoul, Republic of Korea; ^3^KU Center for Animal Blood Medical Science, Konkuk University, Seoul, Republic of Korea

**Keywords:** adverse event, bovine serum albumin, hypersensitivity, lyophilized platelet product, platelet transfusion

## Abstract

**Introduction:**

The present study was designed to evaluate the safety of substances generally used in the preparation of lyophilized platelet products (LPPs) because the possibility of an immune response to bovine serum albumin (BSA) was considered high when using previously described technology.

**Methods:**

An intradermal skin test, followed by a drug provocation test, was conducted to observe adverse events and identify the substances responsible for an immune response. Five male beagles (2 years old) weighing 12–14 kg were used. The dogs were clinically healthy and had no history of medication use. An intradermal skin test was conducted with each substance [i.e., 4-(2-hydroxyethyl)-1-piperazine ethanesulfonic acid, sodium chloride, potassium chloride, sodium bicarbonate, theophylline, trehalose, and BSA] used in the conventional freeze-dry method.

**Results:**

In the intradermal skin test, three dogs tested positive at the BSA injection site and showed clinical signs after the intradermal injection, including nausea and vomiting. For the drug provocation test, all dogs received two intravenous injections of an LPP buffer solution. The initial injection was devoid of BSA, whereas the subsequent injection contained BSA. The three dogs that had reacted to BSA in the intradermal skin test exhibited adverse events such as lethargy, vomiting, and nausea immediately after intravenous injection of the LPP buffer containing BSA. All dogs recovered uneventfully after symptomatic treatment in both tests.

**Discussion:**

The high incidence and severity of type I hypersensitivity reactions observed in this study suggested that BSA is unsuitable as a component of canine LPP.

## 1 Introduction

Extensive research has been conducted on platelet lyophilization in human and veterinary medicine to address the challenges associated with collecting and preserving fresh platelets and enhancing their accessibility for platelet transfusion (1–4). Lyophilized platelets have several advantages, including an extremely long shelf life and convenient storage and transportation. However, at temperatures below 20°C, the platelets become activated, and the membrane structure undergoes irreversible deformation, such as transitioning from a discoid to a spherical shape, thereby activating filopodia and secreting α-granules (1, 2). To minimize structural disintegration in the freeze-dried state, protective substances such as paraformaldehyde or saccharides are used to prevent impairment of the intrinsic structural and functional properties of platelets (3). Saccharides such as trehalose, dextrose, and polysucrose-400 are commonly used.

Albumin, a simple biological protein predominantly found in blood plasma, is commonly involved in preventing platelet aggregation during the drying and rehydration processes. Moreover, it has a significant role in the lyophilization process by serving as a bulking agent and stabilizing the platelets (3). In studies published to date (3–5), heterologous albumin, such as human or bovine serum albumin (BSA), has been used because of the scarcity and availability of canine serum albumin. BSA is a water-soluble protein derived from cows. In various laboratory experiments, BSA has generally been used as a protein concentration standard and added to cell culture media to function as a stabilizer or to support cell growth (6). The protective mechanism remains unclear, although BSA is known to improve the post-freezing recovery and survival of lyophilized cells by increasing the glass transition temperature of the sample, and it is widely used as an extracellular buffer in the freeze-drying of cells (7–9).

However, the most significant concern in using BSA for canine lyophilized platelets is the potential to induce an immune response in recipients, owing to species differences. In veterinary medicine, investigators have reported that BSA could be immunogenic in dogs, thereby being unsuitable for therapeutic use (10, 11). Ohmori et al. (11) described a potential hypersensitivity reaction to BSA in dogs and the relationship between BSA in intravenous medications and beef ingestion. Therefore, the use of BSA in the lyophilization of platelets may induce immune responses such as immediate or delayed hypersensitivity reactions, which can be life-threatening in critically ill patients.

However, to date, most studies on lyophilized platelets have used BSA as a substitute for canine serum albumin, owing to the limited availability and high cost of canine-specific albumin. Despite its common use, persistent concerns regarding the immunogenicity of BSA prompted our hypothesis that a serious immune response could be induced by the BSA components in lyophilized canine platelets. This study was designed to evaluate the *in vivo* safety of materials commonly used in the preparation of canine lyophilized platelet products (LPPs), including BSA. The objectives of this study were (4) to comprehensively evaluate the safety and commercial viability of existing laboratory-produced LPP in dogs while assessing the potential hazards associated with using BSA for canine platelet lyophilization, and (12) to investigate the feasibility and necessity of safer alternatives for BSA in canine lyophilized platelet production.

## 2 Materials and methods

### 2.1 Animals

Five 2-year-old male beagles weighing 12–14 kg were used. The dogs were clinically healthy and were not on any medications that could influence immune responses. They were routinely fed beef-based dry food and exhibited good tolerance without any apparent adverse reactions. The results of the blood tests, including complete blood counts, serum electrolytes, and chemistries, were within the reference ranges for all dogs. This study was approved by the Institutional Animal Care and Use Committee of Konkuk University (Seoul, Republic of Korea; approval number: KU21059).

### 2.2 Study design and classification of the experimental groups

The present study was designed and conducted to evaluate the safety of each substance frequently used in the production of canine LPPs. 4-(2-Hydroxyethyl)-1-piperazine ethanesulfonic acid (HEPES), sodium chloride (NaCl), potassium chloride (KCl), sodium bicarbonate (NaHCO_3_), theophylline, trehalose, dextrose, polysucrose-400, and 5% BSA levels were evaluated. These objects were injected intradermally and later intravenously into healthy beagle dogs, and the dogs were monitored for clinical signs indicating an immune response.

### 2.3 Intradermal skin test

Each constituent of the buffer (i.e., HEPES, NaCl, KCl, NaHCO_3_, theophylline, trehalose, dextrose, polysucrose-400, and 5% BSA) was used for intradermal skin testing (IDST). IDST was administered on the lateral thorax. Hair was gently removed using electric clippers without using any chemical agents, and the injection sites were marked to ensure a minimum distance of 2 cm between each site. Using a 26-gauge needle attached to a disposable 1-mL syringe, the injections were administered at a volume of 0.05 mL of sterile saline (i.e., negative control), 0.05 mL of histamine phosphate at a concentration of 1:100,000 (i.e., positive control), and all individual buffer components diluted in concentration of 1:1,000, after proper preparation. The test sites were evaluated at 15 min and 30 min postinjection while ensuring the prevention of any interference or damage by the animals.

The reactions were scored by assigning a score of 0 to the saline injection and a score of 4+ to the positive control. Subjective assessments were used to compare the allergen responses between the two control groups. A reaction rating of 2+ or higher was deemed potentially significant and required a thorough correlation with the injected material.

### 2.4 Drug provocation test (intravenous injection)

The drug provocation test was carried out by intravenous administration of the LPP buffer solution with or without the constituent suspected of inducing reactions during the IDST. If the dogs exhibited skin reactions after IDST, drug provocation testing proceeded after complete remission of the skin rash (i.e., 1 month after IDST).

Before the intravenous injection, a 22-G intravenous catheter was placed in the left cephalic vein of each dog. The negative control solution was administered using an LPP buffer solution without the suspected material. After injecting the negative control, the LPP buffer containing every component in a volume equivalent to the therapeutic dose of LPP (3.3 × 10^9^ PLTs/kg) was slowly injected intravenously into each dog over 15 min. The intravenous injection was immediately ceased in the event of any adverse reactions, and the symptomatic dog received appropriate treatment, as required. All dogs were monitored for 14 days after the test.

### 2.5 Pre-and post-test examination

Physical examination: A thorough physical examination, consisting of general observation of behavior and level of consciousness, evaluation of mucous membrane color, and capillary refill time, was conducted for each dog before and after the intradermal and intravenous injections. Vital signs, which included systemic arterial pressure (reference range: 90–140 mmHg), respiratory rate (reference range: 15–30 bpm), pulse rate (reference range 80–160 bpm), and rectal temperature (reference range: 38–39°C), were monitored and recorded. The shock index, defined as heart rate/systolic blood pressure, was evaluated before and after the intravenous injection to evaluate any occult or ongoing shock (11). In case of any life-threatening adverse events, the injection was discontinued, and supportive treatment was provided, as required.

Clinical Signs: In the present study, the dogs were observed for a certain period after each test. The dogs were closely monitored for clinical signs indicating adverse reactions such as changes in level of consciousness, fever, nausea, vomiting, edema, and other signs of shock. The dogs were observed for 24 h and 2 weeks after IDST and after a single intravenous injection, respectively.

Hematological analysis: A complete blood count test was conducted for each dog using a flow cytometry hematology system (ProCyte Dx; IDEXX Laboratories, Inc., Westbrook, ME, United States). The platelet, red blood cell, and white blood cell concentrations were analyzed as a pretest examination. Serum biochemistry results were not analyzed.

Naranjo indicator: The Naranjo Algorithm, also known as the Adverse Drug Reaction Probability Scale, utilizes a set of approximately 10 causality assessment criteria (13). These criteria are scored individually. The total score determines the degree of causality between an identified clinical event and drug administration. The interpretation of the scores is as follows: a total score of 9 or higher indicates a definite causal relationship, a score between 5 and 8 suggests a probable causal relationship, a score between 1 and 4 indicates a possible causal relationship and a score of 0 or lower implies a doubtful causal relationship. After the experiment, all dogs were scored using the Naranjo indicator.

### 2.6 Statistical methods

The data obtained were subject to descriptive statistical analysis including the calculation of absolute and relative frequencies and means using the Microsoft Office Excel 2013 software (Microsoft, United States of America).

## 3 Results

**Figure 1:**
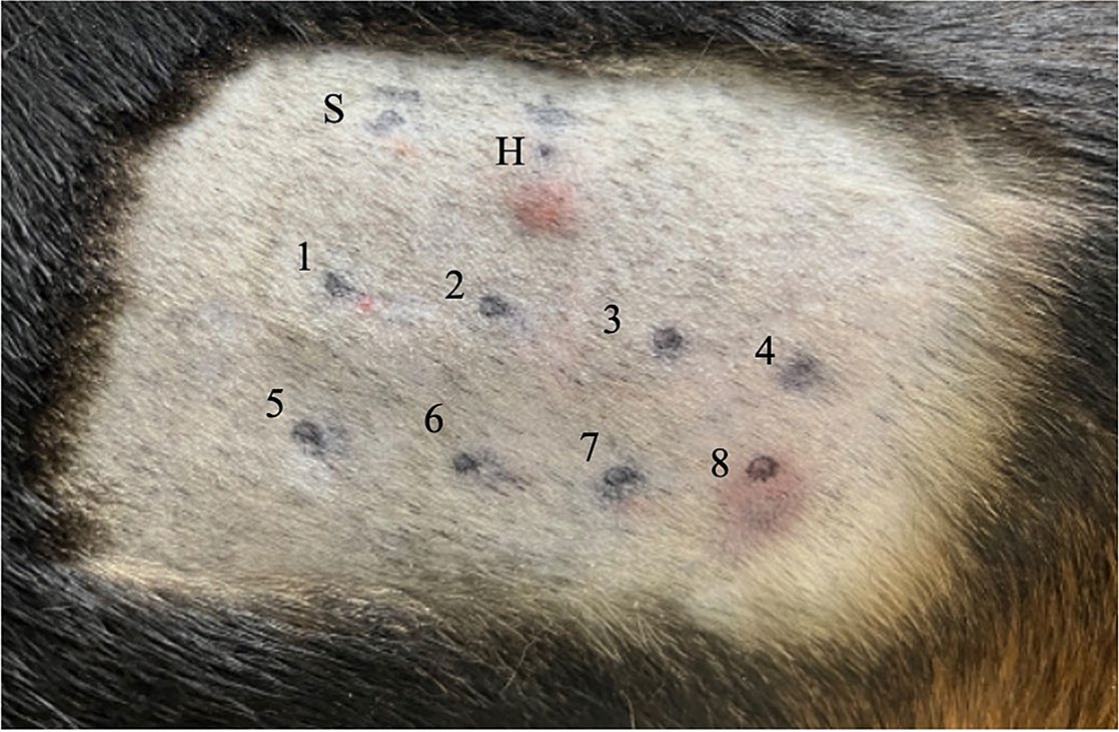
Intradermal skin test results for Beagle 1. Each injection site is indicated as follows: site S is injected with 0.9 sterile saline (i.e., negative control); site H, 0.05 mL of 1:100,000 histamine phosphate (i.e., positive control); site 1, NaCl; site 2, KCl; site 3, NaHCO_3_; site 4, theophylline; site 5, trehalose; site 6, dextrose; site 7, polysucrose-400; and site 8, BSA. A positive reaction, with a score of 4+, based on visual inspection, has occurred at sites H and 8. NaCl, sodium chloride; KCl, potassium chloride; NaHCO_3_, sodium bicarbonate; BSA, bovine serum albumin.

A summary of the study outcomes is presented in Table 1. Details regarding vital sign records, adverse events, treatment, and scores of each scoring system are provided in the Supplementary Material. Three dogs (3/5; 60%) developed skin reactions after IDST. A positive response was observed at the injection sites of the positive control site (i.e., histamine phosphate) and BSA site. On visual inspection, a score of 4+ was assigned to the positive control and the BSA injection site (Figure 1). Dogs showing positive skin reactions to BSA developed local skin responses and demonstrated systemic reactions, notably occurring after intradermal injection of BSA during the IDST. All three symptomatic dogs had nausea, and two dogs vomited. No remarkable changes occurred in vital signs before and after IDST. Two (2/5; 40%) dogs that did not develop any clinical signs had negative IDST results, except for the positive control site.

**Table 1:** Intradermal skin test scores, clinical signs after BSA IV injection, and the Naranjo scale score in five beagle dogs.

	IDST score*	Clinical signs after IV injection of BSA-containing buffer solution							Naranjo Scale
	BSA injection	Tachycardia	Hypotension	SI	Tachypnea	Collapse	Level of consciousness/ behavior	GI signs	Score/ interpretation
Beagle 1	4+	+	+	1.7	+	+	Collapsed after vocalization, stupor	Defecation, nausea, vomit,	12/“Definite”
Beagle 2	4+	+	−	1.19	+	−	lethargy, weakness	Nausea, vomit, defecation	12/“Definite”
Beagle 3	2+	+	−	1.05	+	−	lethargy, weakness	Nausea, vomit	12/“Definite”
Beagle 4	0	−	−	0.95	−	−	Quiet, alert, responsive	Nausea	9/“Definite”
Beagle 5	0	−	−	1.03	−	−	Bright, alert, responsive	None	4/“Possible”

IDST, intradermal skin testing; BSA, bovine serum albumin; IV, intravenous; SI, shock index; GI, gastrointestinal.

*IDST scoring: 0 is assigned to the negative control site (i.e., saline injection), and 4+ is assigned to the positive control site (i.e., 0.05 mL of 1:100,000 histamine phosphate injection). The skin reaction was assessed by comparing the skin reaction at a site versus that of the control sites. Scores > 2+ are significant. The presence of tachycardia, hypotension, tachypnea, and collapse was denoted by the symbol +, while the absence of these symptoms was indicated by the symbol –.

Four dogs had severe systemic to mild clinical signs after the intravenous injection of a buffer containing BSA. Beagles 1, 2, and 3 had immediate adverse responses, including altered mental status, vomiting, vocalization, defecation, and angioedema shortly after initiation. Thus, the injection was stopped. All three dogs had positive reactions to BSA in IDST. Beagle 4, who had a negative reaction to BSA on IDST, exhibited mild gastrointestinal symptoms such as nausea without vomiting.

Beagle 1 collapsed suddenly with multiple gastrointestinal symptoms such as nausea and vomiting, and the systolic BP dramatically decreased from 154 mmHg to 98 mmHg. The dog’s heart rate increased from 96 bpm to 170 bpm. The shock index was elevated above 1, having risen from 0.62 to 1.7, which indicated the increased likelihood of anaphylactic shock. The administration of the LPP buffer solution was immediately stopped. The dog recovered after treatment with epinephrine (0.01 mg/kg IV), diphenhydramine (2 mg/kg IV), crystalloid bolus (20 mL/kg IV), maropitant (1 mg/kg SC), and famotidine (1 mg/kg IV, slowly over 5 min). Beagles 2 and 3 also exhibited altered mental status, weakness, nausea, vomiting, defecation, and facial angioedema shortly after initiation of the BSA injection. The dogs recovered uneventfully after supportive treatment with antihistamines, antiemetics, gastrointestinal protectants, and intravenous fluid therapy. Beagle 4 exhibited nausea after the injection of BSA-containing buffer; therefore, an antiemetic was administered. Beagle 4 spontaneously recovered without further emergency treatment. Finally, Beagle 5 received an intravenous injection without any adverse events. No other adverse effects were observed in any of the five dogs during the 14-day observation period (Table 1).

The Naranjo indicator was scored for all dogs (Table 2). Scores corresponding to “definite” were obtained for all four reactive dogs, and scores corresponding to “possible” were obtained for the 5th beagle, which did not have clinical symptoms after the intravenous injection (13).

**Table 2:** Naranjo adverse drug reaction probability scale scores in five beagle dogs assessed after the injection of LPP buffer containing BSA.

Adverse Drug Reaction Probability Scale (Naranjo Scale)	Score				
Question	Beagle 1	Beagle 2	Beagle 3	Beagle 4	Beagle 5
1. Are there previous conclusive reports of this reaction?	+1 (Yes)	+1 (Yes)	+1 (Yes)	+1 (Yes)	+1 (Yes)
2. Did the adverse event appear after the suspected drug was administered?	+2 (Yes)	+2 (Yes)	+2 (Yes)	+2 (Yes)	0 (No)
3. Did the adverse reaction improve when the drug was discontinued or a specific antagonist was administered?	+1 (Yes)	+1 (Yes)	+1 (Yes)	+1 (Yes)	0 (Do not know)
4. Did the adverse event reappear when the drug was re-administered?	+2 (Yes)	+2 (Yes)	+2 (Yes)	0 (Do not know)	0 (Do not know)
5. Are there alternative causes (other than the drug) that could, on their own, have caused the reaction?	+2 (No)	+2 (No)	+2 (No)	+2 (No)	+2 (No)
6. Did the reaction reappear when a placebo was given?	+1 (No)	+1 (No)	+1 (No)	+1 (No)	+1 (No)
7. Was the drug detected in blood or other fluids in concentrations known to be toxic?	0 (Do not know)	0 (Do not know)	0 (Do not know)	0 (Do not know)	0 (Do not know)
8. Was the reaction more severe when the dose was increased or less severe when the dose was decreased?	+1 (Yes)	+1 (Yes)	+1 (Yes)	+1 (Yes)	0 (No)
9. Did the patient have a similar reaction to the same or similar drugs in any previous exposure?	+1 (Yes)	+1 (Yes)	+1 (Yes)	0 (No)	0 (No)
10. Was the adverse event confirmed by any objective evidence?	+1 (Yes)	+1 (Yes)	+1 (Yes)	+1 (Yes)	0 (Do not know)
Total score	12	12	12	9	4
Interpretation of Scores	Definite	Definite	Definite	Definite	Possible

LPP, lyophilized platelet product; BSA, bovine serum albumin.

## 4 Discussion

The present study was designed to evaluate the safety of substances generally used for the lyophilization of platelets (3–5, 12) in advance of developing off-the-shelf canine lyophilized platelet products. The results of the current study indicate that BSA, a heterologous protein for dogs, is not suitable for manufacturing canine lyophilized platelet products due to immunological safety concerns.

BSA is an inexpensive and readily available protein that is utilized in various biological products and procedures, such as pediatric vaccines, artificial insemination, tissue adhesives, hemostatic agents, and anticancer nano-delivery systems (14–18). Although only a small amount that is considered safe is used, cases of anaphylaxis are repeatedly being reported in humans. In 2008, Pagán et al. (14) reported a case of anaphylactic reaction in a 30-year-old female who underwent standard artificial insemination. The skin prick test, immunoglobulin E (IgE) results, and sodium dodecyl-sulfate polyacrylamide gel electrophoresis IgE immunoblotting strongly suggested that BSA present in the insemination culture medium could be the origin of the anaphylactic reaction. De Silva et al. (18) investigated patients with cow milk allergies who developed anaphylaxis in response to vaccines in Sri Lanka. Based on the findings of the aforementioned study, BSA in vaccines caused allergic reactions in children with cow’s milk allergy because 76.5% of children in the cohort were sensitized to BSA, based on BSA-specific IgE results.

Hypersensitivity reactions to oral, intradermal, intramuscular, and intravenous BSA injections have been reported in dogs (10, 19–23). Based on these studies, BSA is inadequate for therapeutic use in canines. Mosley et al. (10) aimed to evaluate the potential of BSA as a cost-effective and readily available alternative treatment option to replace human serum albumin, which is commonly used in hypoalbuminic dogs. According to an internet-based survey conducted by Yozova et al. (24), human serum albumin has been reported to be utilized more frequently than canine albumin by 1,134 veterinarians across 42 countries, despite the potential risks associated with adverse effects. This experimental study revealed adverse reactions in healthy dogs after two injections of BSA. With an intravenous dose of 500 mg/kg BSA, one dog (1/10; 10%) developed immediate reactions such as mild urticaria and pruritus after the first injection. After the second injection in two dogs, a severe type I hypersensitivity reaction occurred in one (50%) of the dogs. Ohmori et al. (19) report that BSA included in vaccines for dogs can function as an allergen, and allergic reactions can occur in dogs postvaccination. The findings of the aforementioned studies suggest that BSA is inadequate as a therapeutic agent for dogs, and it may trigger an allergic response when used as a vaccine. Anaphylaxis caused by vaccination is rare; therefore, veterinarians should always be cautious because vaccination can lead to life-threatening cardiovascular or respiratory manifestations (10, 21, 22).

In this study, an intradermal skin test was conducted to identify the potential immunogenic responses to each component. Intradermal skin and skin prick tests are widely employed in human medicine because they are practical, convenient, and reliable. IDST serves as the primary means of assessing sensitization to suspected drugs (25, 26). IDST is commonly used in veterinary medicine, particularly for the diagnosis of IgE-mediated diseases. Individual allergens were injected intradermally (generally, 0.05 mL in volume) into the IDST site. In the interpretation of IDST, skin reaction scores >2+ are considered potentially significant and should be interpreted in combination with clinical signs and history (25, 26).

In this study, three dogs with skin reaction scores exceeding 2+ for BSA exhibited nausea after BSA injection during the IDST. Among these dogs, two developed a substantial skin reaction with a score of 4+ in response to BSA and subsequently experienced nausea and vomiting. These dogs were clinically healthy before IDST and had no history of disease or medication. Given that gastrointestinal symptoms manifested in three dogs with positive responses to BSA on IDST in this study, BSA is highly likely to function as a substantial allergen in dogs.

Adverse reactions to medication are diagnosed as follows: Medical histories and clinical signs are evaluated when a drug hypersensitivity reaction is suspected. Skin or laboratory tests are performed when the possibility of drug hypersensitivity exists based on a patient’s history and symptoms. If available, *in vitro* tests can be added (27, 28). Positive results from skin or laboratory tests indicate that the suspected drug can cause hypersensitivity. Provocation tests are indicated when the triggering drug cannot be identified by skin or laboratory tests (27, 28).

In this study, intravenous injections of LPP buffer solution with or without BSA were used for the drug provocation test. Three dogs that had a positive skin response in the previous IDST thereafter exhibited acute systemic clinical signs such as altered mental status, weakness, vomiting, and facial angioedema after the injection of BSA-containing buffer solution. Furthermore, among the two dogs that did not exhibit any significant response during IDST, one dog experienced an immediate onset of nausea on the initiation of an intravenous injection of the buffer solution containing BSA. In a previous study by Mosley et al. (10) in which a BSA concentrate was injected to evaluate its clinical use in 10 healthy dogs, acute reactions such as severe anaphylactic reaction (type I hypersensitivity), mild urticaria, and pruritus were observed in three dogs after intravenous injection. The reaction to BSA injection was similar to that observed in this study.

Hypersensitivity can be classified based on the cells involved and the speed at which the reaction occurs. Type I hypersensitivity manifests in two distinct stages: sensitization and the subsequent effect stage. Re-exposure of a pre-sensitized host to the antigen can lead to acute reactions characterized by vasodilation and smooth muscle contraction. In the present study, an acute immune response occurred immediately after the BSA injection, and the symptomatic dogs exhibited typical signs such as edema, nausea, vomiting, and changes in the level of consciousness. Notably, the dogs in this study maintained a regular dry food diet primarily consisting of beef and exhibited good tolerance without any significant adverse reactions. We hypothesize that this diet may have contributed to the sensitization phase, potentially influencing the observed immune responses. Therefore, the likelihood of type I hypersensitivity is plausible. These immune responses resolved after emergent treatment, including epinephrine and antihistamine administration.

The Naranjo algorithm, also called the Naranjo Scale, is a questionnaire developed by Naranjo et al. to evaluate the probability of adverse drug reactions in human medicine (13). Naranjo algorithm consists of 10 questions that are answered by “Yes,” “No,” or “Do not know.” Different points were assigned to each answer, and the total score was assessed. The 10 questions include the relationship between drugs and the occurrence of adverse drug events such as the administration and the occurrence of adverse drug events; the patient’s reaction after the discontinuation or re-administration of the drug; evaluation of the possibility that sources other than the drug and the patient’s underlying disease may be involved; and items such as the presence or absence of a similar reaction to the drug, the response to administration of placebo, measurement of drug concentration, and the presence or absence of an objective test to support adverse drug events. The Naranjo algorithm is known to have high predictive accuracy for true adverse drug reaction determination among suspected cases (13, 29). The Naranjo scale is occasionally utilized for veterinary patients when a suspected drug adverse reaction arises, owing to the absence of an equivalent scale in veterinary medicine (30–32). Given the considerable suspicion of type I hypersensitivity induced by BSA in our study and the absence of a specific drug adverse reaction probability scale designed for veterinary medicine, we employed the Naranjo scale, a commonly utilized tool in human medicine. In the current study, the Naranjo scale score was “definite” for all four symptomatic dogs, thereby suggesting a high probability of BSA acting as a significant allergen. In the present study, significant clinical signs developed after the intradermal intravenous injection of a buffer containing BSA. Considering the results of this study and the Naranjo Adverse Drug Reaction Probability Scale, which scored “definite” in four dogs (4/5, 80%), the inclusion of BSA in canine LPP buffer poses a high risk of inducing type I hypersensitivity reactions. Thus, it is recommended that BSA should be avoided in dogs, particularly when preparing lyophilized platelets. BSA should be excluded from LPP buffer components to prevent anaphylactic reactions in patients receiving lyophilized platelet products.

This study had some limitations. First, we did not conduct *in vitro* tests such as serological testing. If serological testing had been performed, dogs with no or mild clinical signs may have had an increase in serum IgE levels. Skin and serum IgE tests are complementary; therefore, applying both tests simultaneously should be considered in studies investigating sensitized populations (33). We could not proceed with specific IgE testing because of the lack of serum samples. Further studies that include IDST and serum IgE testing are warranted. Finally, the small sample size in the present study may have resulted in type 2 errors.

In conclusion, BSA appears to be an inappropriate component for manufacturing canine lyophilized platelet products because of its potential immunogenicity due to species differences, as indicated in the present study. Although BSA has been commonly used for the stabilization and restoration of appropriate platelet morphology after the resuspension of freeze-dried platelets, the results of the present study suggest that an alternative material to substitute for BSA is required for manufacturing canine lyophilized platelets. In addition, considering the high risk of hypersensitivity reactions to BSA in dogs, caution is warranted for any product containing BSA intended for use in dogs, including vaccines, drugs, tissue adhesives, and hemostatic agents.

## Data availability statement

The raw data supporting the conclusions of this article will be made available by the authors, without undue reservation.

## Ethics statement

The animal study was approved by the Institutional Animal Care and Use Committee of Konkuk University. The study was conducted in accordance with the local legislation and institutional requirements.

## Author contributions

H-JC: Conceptualization, Data curation, Writing – original draft. M-YK: Conceptualization, Investigation, Writing – review & editing. H-JH: Methodology, Supervision, Writing – review & editing.
