# Supplementary material for: Safety evaluation of a buffer used in the lyophilization of canine platelets: type I hypersensitivity reaction in dogs caused by bovine serum albumin
Source: Front Vet Sci. 2024 Feb 12;11:1344037. doi: 10.3389/fvets.2024.1344037 (PMC10895064; doi:10.3389/fvets.2024.1344037)
Supplement: Supplementary file 1 [file Table_1.DOCX]

Supplementary Material

Safety evaluation of a buffer used in the lyophilization of canine platelets: Type I hypersensitivity reaction in dogs caused by bovine serum albumin

Hee-Jae Choi^1^, Mu-young Kim^2^, Hyun-Jung Han^1,3^ *

*** Correspondence:**Hyun-Jung Han
Email: [ab1234@konkuk.ac.kr](mailto:ab1234@konkuk.ac.kr)

# Supplementary Table

Supplementary Table 1. Vital signs and mean blood pressure before the IV injection of BSA-containing buffer solution in five beagle dogs. In cases of adverse events, the clinical signs and treatment were recorded.

|  | **Before IV injection (baseline)** | | | | |  | **Post IV injection** | | | | | |
| --- | --- | --- | --- | --- | --- | --- | --- | --- | --- | --- | --- | --- |
|  | Heart rate (HR) (bpm) | Respiratory rate (RR) (breaths/min) | Rectal temperature (°C) | Mean blood pressure (BP), systolic/diastolic(mean arterial) (mmHg) | Level of consciousness |  | HR (bpm) | RR (breaths/min) | Rectal Temperature (°C) | mean BP, systolic/diastolic (mmHg) | Adverse event | Treatment |
| Beagle 1 | 96 | 24 | 38.8 | 154/127 (137) | BAR |  | 170 | 60 | 39 | 98/52 (74) | Collapse, nausea, vomiting, defecation, vocalization, angioedema | Epinephrine, crystalloid shock dose ¼, antiemetics, GI protectants, antihistamine |
| Beagle 2 | 144 | 36 | 38.6 | 153/102 (131) | BAR |  | 172 | 66 | 38.6 | 144/107 (123) | Decreased level of consciousness, weakness, nausea, vomiting, defecation, angioedema | Antiemetics, GI protectants, antihistamine, IV fluid |
| Beagle 3 | 136 | 36 | 38.8 | 155/128 (143) | BAR |  | 160 | 60 | 38.9 | 151/118 (125) |  |  |
| Beagle 4 | 150 | 30 | 39 | 138/70 (101) | BAR |  | 148 | 30 | 39 | 144/98 (124) | Mild GI signs | Maropitant (1 mg/kg, SC) |
| Beagle 5 | 141 | 36 | 39.1 | 142/96 (127) | BAR |  | 142 | 36 | 39.1 | 137/78 (108) | None | None |

IV, intravenous; BAR, bright, alert, and responsive; GI, gastrointestinal; SC, subcutaneous
